# Dalangtan Playa (Qaidam Basin, NW China): Its microbial life and physicochemical characteristics and their astrobiological implications

**DOI:** 10.1371/journal.pone.0200949

**Published:** 2018-08-01

**Authors:** Ting Huang, Ruicheng Wang, Long Xiao, Hongmei Wang, José M. Martínez, Cristina Escudero, Ricardo Amils, Ziye Cheng, Yi Xu

**Affiliations:** 1 State Key Laboratory of Geological Process and Mineral Resources, Planetary Science Institute, China University of Geosciences, Wuhan, Hubei, China; 2 State Key Laboratory of Biogeology and Environmental Geology, China University of Geosciences, Wuhan, Hubei, China; 3 Space Science Institute, Macau University of Science and Technology, Macau, China; 4 Centro de Biología Molecular “Severo Ochoa” (UAM-CSIC), Madrid, Spain; 5 Centro de Astrobiología (CSIC-INTA), Torrejón de Ardoz, Madrid, Spain; Swedish Museum of Natural History, SWEDEN

## Abstract

Dalangtan Playa is the second largest salt playa in the Qaidam Basin, north-western China. The hyper saline deposition, extremely arid climate and high UV radiation make Dalangtan a Mars analogue both for geomorphology and life preservation. To better understand microbial life at Dalangtan, both culture-dependent and culture-independent methods were examined and simultaneously, environment conditions and the evaporitic mineral assemblages were investigated. Ten and thirteen subsurface samples were collected along a 595-cm deep profile (P1) and a 685-cm deep profile (P2) respectively, and seven samples were gathered from surface sediments. These samples are composed of salt minerals, minor silicate mineral fragments and clays. The total bacterial cell numbers are (1.54±0.49) ×10^5^ g^−1^ for P1 and (3.22±0.95) ×10^5^ g^−1^ for P2 as indicated by the CAtalyzed Reporter Deposition- Fluorescent in situ Hybridization (CARD-FISH). 76.6% and 75.7% of the bacteria belong to Firmicutes phylum respectively from P1 and P2. In total, 47 bacteria and 6 fungi were isolated from 22 subsurface samples. In contrast, only 3 bacteria and 1 fungus were isolated from 3 surface samples. The isolated bacteria show high homology (≥97%) with members of the Firmicutes phylum (47 strains, 8 genera) and the Actinobacteria phylum (3 strains, 2 genera), which agrees with the result of CARD-FISH. Isolated fungi showed ≥98% ITS1 homology with members of the phylum Ascomycota. Moisture content and TOC values may control the sediments colonization. Given the deliquescence of salts, evaporites may provide refuge for microbial life, which merits further investigation. Halotolerant and spore-forming microorganisms are the dominant microbial groups capable of surviving under extreme conditions. Our results offer brand-new information on microbial biomass in Dalangtan Playa and shed light on understanding the potential microbial life in the dried playa or paleo-lakes on Mars.

## Introduction

Mars today presents a cold, hyper-arid environment which is very challenging for life survival. However, it is well-proven that in the past Mars had abundant water on its surface [1–3] and a biosphere might even have existed in the fluvial-lacustrine environments during the post-Noachian period (from 3.7 billion years ago to present epoch) [4]. As Mars changed to a cold and arid planet, almost all the water on its surface was lost, while the remaining water was conserved in water ice [5], hydrated minerals [6] and underground briny aquifers [7]. Current liquid brines in shallow subsurface were considered as one of the sources for formation of gullies on Mars [8, 9]. The Recurring Slope Linea (RSL) [10] was proposed as evidence for recurring brine on Mars surface during warm seasons [11], although other hypotheses are also being considered [12]. These observations provided a basis for the possibilities of life existence in saline environments and subsurface aquifer systems on Mars. The conditions referred to above must be considered critical research sites for life detection on Mars [13, 14].

There are four ways to acquire information about potential Martian life traces: in-situ analyses accessed by lander and rover missions on Mars, analysis of identified Martian meteorites, ground/ space simulation of Martian conditions in laboratories and terrestrial analogue studies. Landed missions equipped with instruments such as the MER Rovers (NASA, 2003) [15, 16] and the Curiosity Rover (NASA, 2011) [17, 18] analyzed the physicochemical characteristics of materials on Mars surface and shallow subsurface to detect organic matter and other potential life sources. However, without samples sent back to Earth, landing missions to unique areas provide interesting but limited information. Martian meteorites have conserved information about Mars materials, its geological history [19, 20] and organic signatures [21]. Whereas, it is extremely difficult to identify life traces in Martian meteorites mainly due to the contamination origin of the organic materials detected from them [22, 23]. Simulation chambers mimic Martian conditions and produce a sample analogue to investigate changes in minerals, biosignatures and microorganisms [24, 25]. Nevertheless, space provides a mixture of extreme environmental parameters not easily simulated in particular due to physical properties of the facilities [26].

In comparison, Mars-like conditions on Earth, to some extent, are considered promising research models for Mars, such as hyper arid desert [27], low-pH environments [28], permafrost [29] and hydrothermal system [30, 31]. In this regard, terrestrial analogues can provide a much easier sample collection for analysis. A large body of research has been done in terrestrial super arid and hyper saline conditions to understand by comparison with Mars environments, including Atacama Desert [27], the Great Salt Plains of Oklahoma [32], and Chott el Gharsa in Tunisia [33]. Despite the harsh conditions in those environments, lives including both prokaryotes (archaea and bacteria) and eukaryotes (algae and fungi) are detected [34, 35]. Also great efforts have been laid on the detection of limits for life in many different physicochemical conditions, i.e. pH, temperature, water activities, radiation and pressure [36, 37]. Microbial explorations in these hyper arid and hyper saline conditions enhance our understanding of the life diversity and provide information for putative habitats on Mars.

The Qaidam Basin in north-western China is an inland basin developed since the Jurassic period [38]. With the average elevation of 2800 m in the central part [39], the Qaidam Basin has an annual precipitation ranging from 16 to 190 mm and an annual evaporation varying between 1974 and 3183 mm [40]. According to data from 1980 to 2011 at Mangya metrological station in the Basin, the monthly average relative humidity (RH) ranged from 23% to 26%, among which, RH of June (in summer season) was 32% [41]. The UV radiation is very strong across the region and the annual average temperature is about 2–4 °C [42]. It is covered mostly by desert, desert steppes, exposed bedrock and lacustrine deposits [43]. Mars-like conditions such as aeolian landforms and playas are widespread in the basin [44]. Considering the Martian characteristics, the Qaidam Basin has been regarded as a promising scientific research site for Mars analogue studies [45, 46].

Dalangtan playa is a special area among the natural landforms with high potentiality to serve as Martian analogue in the Qaidam Basin. It is the second largest salt playa (~ 210 km^2^), and one of the most arid regions in China. Dalangtan region originated from an ancient dried out lake and nowadays is covered by salt deposits with wide distribution of halite and gypsum [47]. Moreover, aeolian landforms are widely spread due to strong impact of wind forces. Because of its geological characteristics and physical conditions, Dalangtan Playa could be considered as an excellent terrestrial analogue for the study of Martian evaporites and life occurrence.

Several studies have been conducted at Dalangtan Playa since 1970s on its geological history and salt resources [48, 49]. Recently, evaporite minerals attract more attention due to their significance in understanding Martian environmental conditions [50]. Together with in-situ observation in the Qaidam Basin and experimental research, subsurface hydrous sulfates on Mars formed during past high obliquity periods (>45°) are indicated to maintain mid-to-high degrees of hydration, even until the present epoch [51]. By comparing mineralogy and environmental conditions of Dalangtan Playa with analogous conditions on Mars, it has been proposed that the hydrated sulfates observed in the surface by remote sensing, especially widespread kieserite (MgSO_4_·H_2_O), are likely weathering products rather than pristine deposits [52]. From a microbial perspective, 8 isolates of bacteria belonging to 6 genera (*Virgibacillus*, *Oceanobacillus*, *Halobacillus*, *Terribacillus*, *Streptomyces* and *Acinetobacter*) were isolated from Dalangtan Playa [53]. Bacterial fatty acids, archaeal acyclic diether and tetraether membrane lipids have also been detected. A dominance of Gram-positive bacteria and halophilic archaea in this hypersaline ecosystem have been described [54]. These results have enhanced our understanding of Dalangtan Playa as a Mars analogue, but knowledge about microbes living in this extreme environment is very limited, which subsequently impedes our understanding about astrobiology on Mars. Any recovery of microorganisms from the extreme conditions and their correlation with environmental conditions will offer valuable information for astrobiological investigation.

The purpose of this study is to investigate the microbial diversity and mineral assemblages in the surface and subsurface of Dalangtan Playa. The extremely low moisture content (0.08%), low TOC (0.03%) and the high salinities (50%) are very challenging for life survival when compared with other studied terrestrial sites. The results of this study will provide information for life exploration on Mars, especially for ancient lake conditions.

## Materials and methods

### Geological setting and sediment sampling

**Fig 1:**
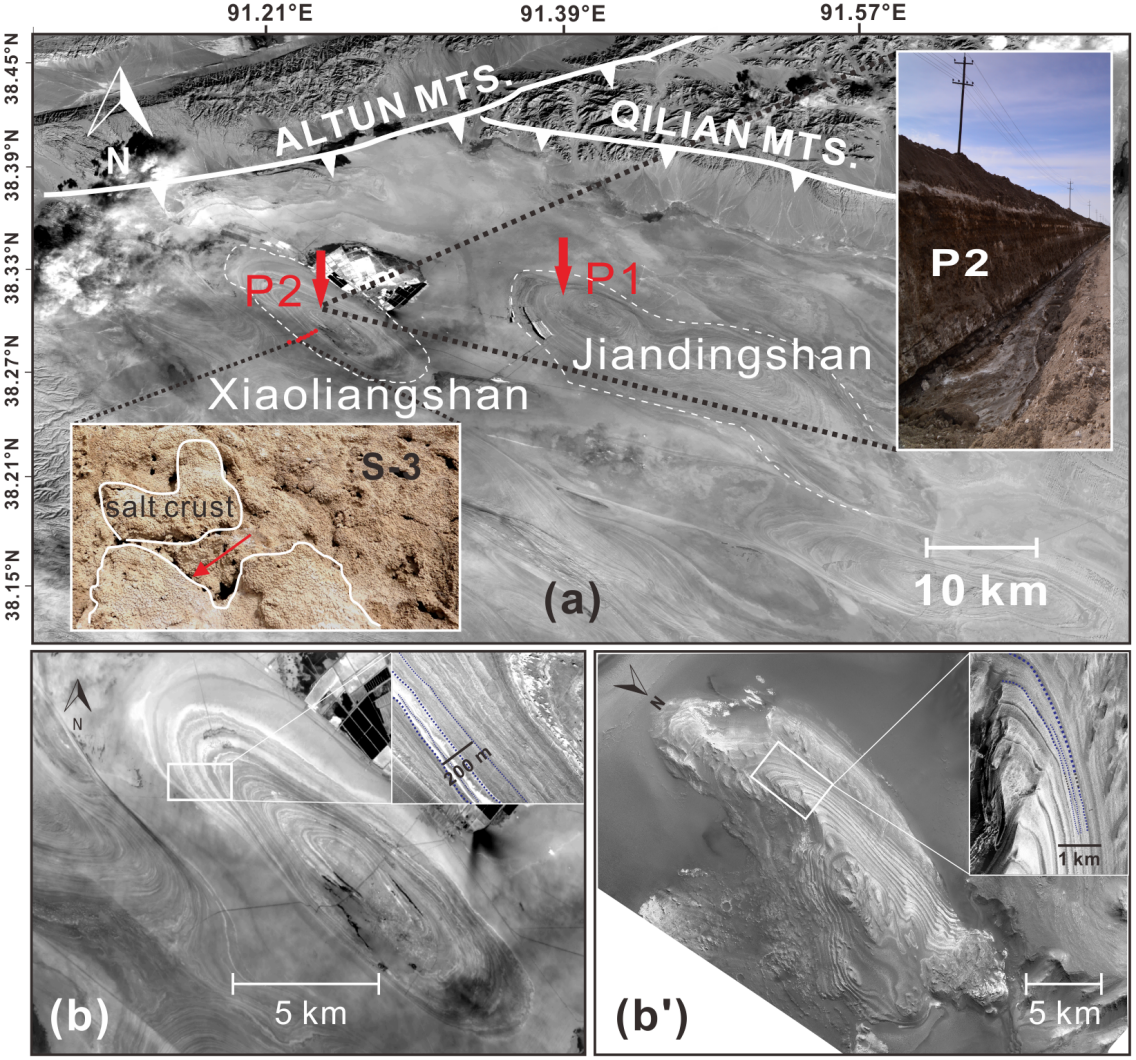
Studying site and its comparison with Mars layered deposits. (a) Map of Dalangtan Playa, red arrows indicate sampling locations for profiles, recently excavated by brine mining activities, P1 and P2, red points indicate the sites for surface sampling, i.e. sample S-1 to S-7 from north-east to south-west. Details of S-3 and P2 are shown. Sample of S-3 was collected under salt crust, pointed by red arrow. (b) Xiaoliangshan anticline, its layered structure (zoomed in white box) and its comparison to Mars interior layered structure in Juventae Chasma (b’). Images of a and b were acquired from the U.S. Geological Survey archive (https://www.usgs.gov/). The b’ includes one Context Camera image B10_013600_1756_XI_04S062W, and one High Resolution Imaging Science Experiment image ESP_020470_1755 (top right in b’). These data were obtained from the NASA Planetary Data System (https://pds.nasa.gov/). S-3 and P2 in (a) were acquired by field trips.

Dalangtan Playa is located at the north-western Qaidam Basin rimmed by Altun-Qilian Mountains to the north (Fig 1a). An ancient giant lake developed in the basin since the Jurassic period. The lake expanded and migrated from north-west to south-east in the late Miocene to early Pliocene epoch. During the migration period, the single large ancient lake split into several small secondary lakes due to uneven lakebed and later tectonic, and most of them became playas due to tectonic movements and climate change. Of which Dalangtan area was one of the firstly dried out small lakes [38].

At present, Dalangtan Playa is covered by Quaternary sediments. Evaporites such as halite and gypsum are widespread in this region [55]. According to the results from the U-series disequilibrium dating for the profile of P1, the ages of the gypsum located at 110 cm and 210 cm depth are 65.6±6.2 ka and 79.8±6.6 ka respectively. The age of the gypsum located at 600 cm depth on P2 profile is 123.5±13.0 ka [54].

Our studying sites were in Xiaoliangshan and Jiandingshan anticlines inside the playa (Fig 1a). Xiaoliangshan anticline (Fig 1b) is more like an oval-shaped and layered structure, with a long axis of 19.5 km and a short axis of 5.5 km. The height of Xiaoliangshan is ~60 m. Jiandingshan anticline is irregular in shape and with a height of ~70 m. Alternating bright and dark laminations can be seen in both anticlines through satellite image. Morphologically the laminations are similar to interior layered deposits identified on Mars [56]. A comparison between layered structure of Xiaoliangshan (Fig 1b) and Mars interior layered deposits in Juventae Chasma (Fig 1b’) is shown. The latter is interpreted as sulfate-bearing layers [57]. Similarly, the layered structures of Xiaoliangshan anticline were firstly interpreted as different mineral assemblages, such as phyllosilicates and sulfates [50, 58]. Surface samples were collected along south-east to north-west of Xiaoliangshan anticline, denoted as S-1 to S-7, collected from a depth of 10 cm to 30 cm. Profiles P1 (38°31'53.75"N, 91°34'54.54.68"E) and P2 (38°30'11.62"N, 91°23'53.11"E) were located in Jiandingshan and Xiaoliangshan anticlines respectively. Ten samples collected from a 590-cm deep P1 profile have been designated as P1-37 to P1-590. Thirteen samples were collected along profile P2 with a depth of 685 cm and designated as P2-50 to P2-685 according to the lamination with different colors and textures of the sediments.

Samples were collected in sterile conditions and stored immediately in 50 ml aseptic plastic corning centrifuge tubes under ambient conditions during the field trip, transported to the Geomicrobiology Laboratory (China University of Geosciences, Wuhan) and stored at 4 °C and -20 °C for further analysis. Temperatures during the field trip (20^th^ to 30^th^ of June 2015) were between 7 to 25 °C.

### Physicochemical analysis

An aliquot of 10 g samples was mixed with ultra-pure water with a ratio of 1:2.5 (w/v) and homogenized. Solutions were allowed to sit for 30 minutes and followed by pH measurement (UB-7 DENVER, America) in triplicate. Salinities (salt concentrations) were tested by determining the electrical conductivity (EC) of either a 1:5 or 1:10 sediment dilution with distilled water (ATAGO ATC-20E, Japan) at 25 °C. Moisture contents were the mass loss of original samples after freeze drying (ALPHA 1–2 LD, Martin Christ, Germany) which is calculated via moisture content (%) = (Mass _wet_ -Mass _dried_)/ Mass _wet_.

X-ray diffraction (XRD) analysis was conducted with an X-ray diffractometer (D8-FOCUS, BRUKER Ltd.) with Cu X-ray source and with scan speed of 0.05 sec/step. Data of XRD were analyzed semi-quantitatively with both XPowder (http://www.xpowder.com/) and JADE (http://materialsdata.com/) software.

For total organic carbon (TOC) measurements, approximately 10 g of the sample were dried and ground. The sample was subsequently suspended in 40 ml 4M hydrochloric acid solution for 24 hours. The acidic solution served to break down the carbonates present in the sample. Supernatants of the mixture were discarded after centrifugation. Precipitates were subsequently washed with ddH_2_O for three times. After that, precipitates were frozen dried and ground again. TOC was analyzed with an elemental analyzer (ELEMENTAR, Germany).

### Microbial isolation and identification

Liquid enrichment media and agar plates were used to isolate microorganisms from samples of Dalangtan Playa. Four different media were used in this study, they are synthetic medium MGM [59], R2A (Difco), Marine Broth (Difco) and Arq media (composition described in S1 Table). Salinities of the media were adjusted to 0, 3.5, 10, 18, 21, and 35% by supplemented with different amount of NaCl to recover microorganisms with different salinity requirements. The incubation temperature was set at 25 °C which is close to the temperature during sampling. Except for the 35% salinity media which were incubated at 30°C. Abiotic control experiments were set in parallel under same experimental conditions except the replacement of sample inoculum with sterile water.

Identification of microbial isolates was performed by molecular analysis of 16S rRNA for bacteria and ITS1 for fungi respectively in the conditions described in [60, 61]. Sequencing was conducted by Tianyi Huiyuan Bio-Technique Co. Ltd., Wuhan, China, and the Sequencing Unit of the Astrobiology Center (INTA-CSIC) for bacteria and GenScript, Nanjing, China for fungi. All the sequences from this study have been submitted to the NCBI GenBank database with accession numbers of KY243889 to KY243911 for bacteria and KY775058 to KY775064 for fungi.

The selected representative sequences were aligned and subjected to phylogenetic analysis using the ClustalW program. Maximum Likelihood method was used for the construction of phylogenetic trees (MEGA7) [62]. *Chlamydia trachomatis* and *Saccharomycetes* sp. were served as out groups to root the trees of bacteria and fungi respectively. Distances were calculated using the Kimura method [63]. Maximum Likelihood trees were conducted using Tamura 3-parameter model method with 1000 bootstrap replicates (less than 15 taxa).

Selected isolates were grown in MGM medium at 25 °C at various salinity concentrations ranging from 12%, 15%, 18%, 21% and 24%. Growth was monitored by measuring the optical densities at 600 nm (TU-1800 PERSEE, Shanghai, China) with 24 hours interval. Experiments were performed in duplicates.

An aliquot of 2 μl microbial culture of each strain was mounted onto glass cover slips, which were pretreated with poly-L-lysine for 15 min. After fixation with paraformaldehyde and glutaraldehyde, the samples were sequentially dehydrated through grade series of ethanol followed by critical point drying with a K850X Critical Point Drier (Quorum, UK) [64]. After about 20 s of platinum sputtering treatment, the samples were observed using a Hitachi SU8000 Scanning Electron Microscope (SEM) in the State Key Laboratory of Biogeology and Environmental Geology (CUG, Wuhan).

### Culture independent methods

#### DNA extraction

About 1g sediments were used for DNA extraction. Different methods were used, (i) mechanical lysis by grinding, (ii) mechanical lysis in the presence of liquid nitrogen, (iii) mechanical lysis followed by sonication in the presence of PBS and ethanol (1:1), (iv) filtering was introduced after sonication, both the membranes mounted with sediment samples and supernatant were subjected for subsequent DNA extraction steps, (v) mechanical lysis followed by phenol extraction as described in [65], (vi) PowerMax and PowerSoil (QIAGEN, Inc. USA) commercial extraction kits.

Extracted DNA products were concentrated by Speed-Vac concentrator (Savant, Hicksville, USA). Primers used in this study included (i) 21F (5’AGAGTTTGATCMTGGCTCAG3’) and 1492R (5’TACGGYTACCTTGTTACGACTT3’), S-D-Bact-0341-b-S- 17 (5’CCTACGGGNGGCWGCAG3’) and S-D-Bact-0785-a-A- 21 (5’GACTACHVGGGTATCTAATCC3’) for bacteria, (ii) Arch1R (5’CGGRAAACTGGGGATAAT3’) and Arch1F (5’TRTTACCGCGGCGGCTGBCA3’) for archaea and (iii) 563F (GCCAGCVCYGCGGTAAY) and 1132R (CCGTCAATTHCTTYAART) for eukaryotes. Concentrations of extracted DNA were measured with a nanodrop 1000 spectrometer (Thermo Fisher Scientific Inc., USA) and Qubit 2.0 (Thermo Fisher Scientific Inc., USA). Inhibitors testing was designed as follows: three separate parts of prepared DNA materials (A, boiled fresh *E*. *coli* cells, B, boiled fresh *E*. *coli* cells + targeted DNA products and C, target DNA products) were set for PCR, checked by agarose gel electrophoresis, stained with ethidium bromide, and visualized by UV light.

#### CARD-FISH method to detect microorganisms

Samples were fixed with 4% formaldehyde and stored at -20°C in ethanol: PBS (1:1) until further processing. CARD-FISH was performed following the protocols described in [66] with a minor modification: tyramide signal amplification was carried out for 45 min at 46 °C. Hybridizations were performed with 5’-HRP-labled oligonucleotide probes (Biomers, Ulm, Germany) and stringencies were regulated by adjusting formamide (FA) and NaCl concentration in hybridization and washing buffer respectively: EUB388 I-III mix [67, 68] and LGC354a, b [69] 35% FA (v//v), 0.08M NaCl; ARC915 [70] 20% FA (v/v), 0.225M NaCl. Samples were counterstaining with DAPI (4’,6-diadimino-2-phenylidole) or Syto9 (Thermo Fisher Scientific, USA) as manufacturer recommended and covered with a Vectashield (Vector Laboratories, Burlingame, USA): Citifluor (Citifluor, UK) (1:4) mixture. CARD-FISH detection of microorganisms was done by epifluorescent microscopy (Zeiss-Axioskop, BIOSURPLUS EAST, USA). Each sample was prepared in triplicates, and each 15 visual fields were checked per triplicate. Samples were imaged by using confocal laser scanning microscope (LSM510 vertical, LSM Tech, USA) coupled with a vertical microscope Axio Observer (Carl Zeiss, Germany) and equipped with argon (488/514 nm) and helium and neon (543 and 633 nm) lasers. Images were collected with a 63×/1.4 oil immersion lens.

## Results

### Physicochemical properties and mineral assemblages

The pH values of surface samples varied from 7.95 to 8.90. TOC was generally low with a range between 0.03% and 0.12%. Surface minerals are mainly composed of halite, gypsum and thenardite, with minor detrital grains (Table 1).

**Table 1:** Physicochemical characteristics of surface samples in Dalangtan Playa.

Sample ID	pH	TOC (%)	Mineral assemblage (w/w)
**S-1**	7.97	0.06	Halite 99% and amorphous 1%
**S-2**	8.90	0.03	Halite 51.3%, Thenardite 45.5% and amorphous 3.2%
**S-3**	8.34	0.12	Halite 71.9%, Gypsum 11.4% and Quartz 16.7%
**S-4**	8.30	0.07	Halite 79%, Gypsum 18.5% and amorphous 2.5%
**S-5**	8.50	0.10	Halite 30.2%, Gypsum 34.0%, Microcline 32.1% and amorphous 3.7%
**S-6**	8.27	0.09	Halite 98.1% and amorphous 1.9%
**S-7**	7.95	0.11	Halite 90.4%, Gypsum 6.1%, and Quartz 3.5%

**Fig 2:**
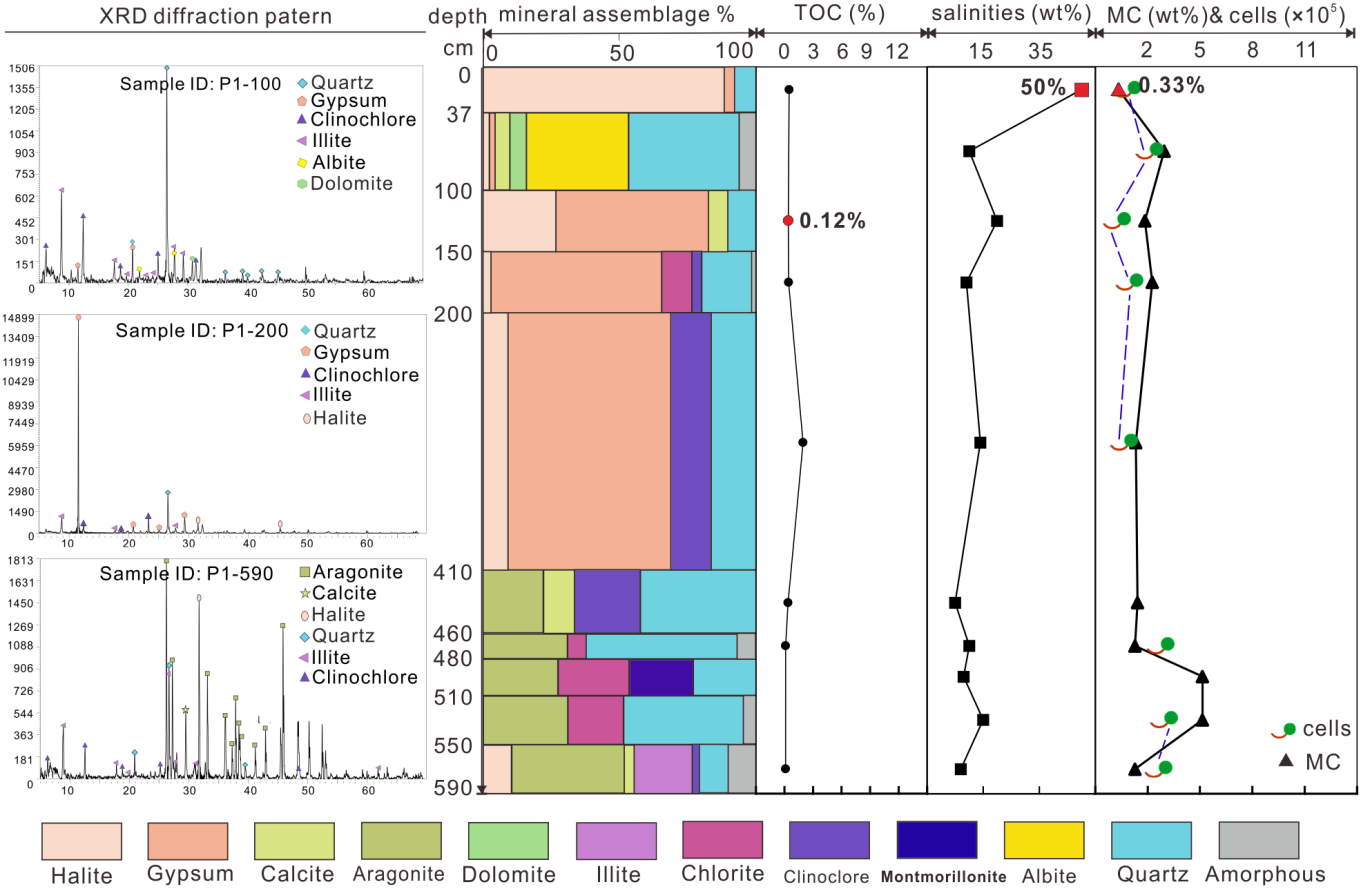
XRD diffraction patterns, mineral assemblage, TOC, salinity, moisture and cell numbers of P1 profile.

**Fig 3:**
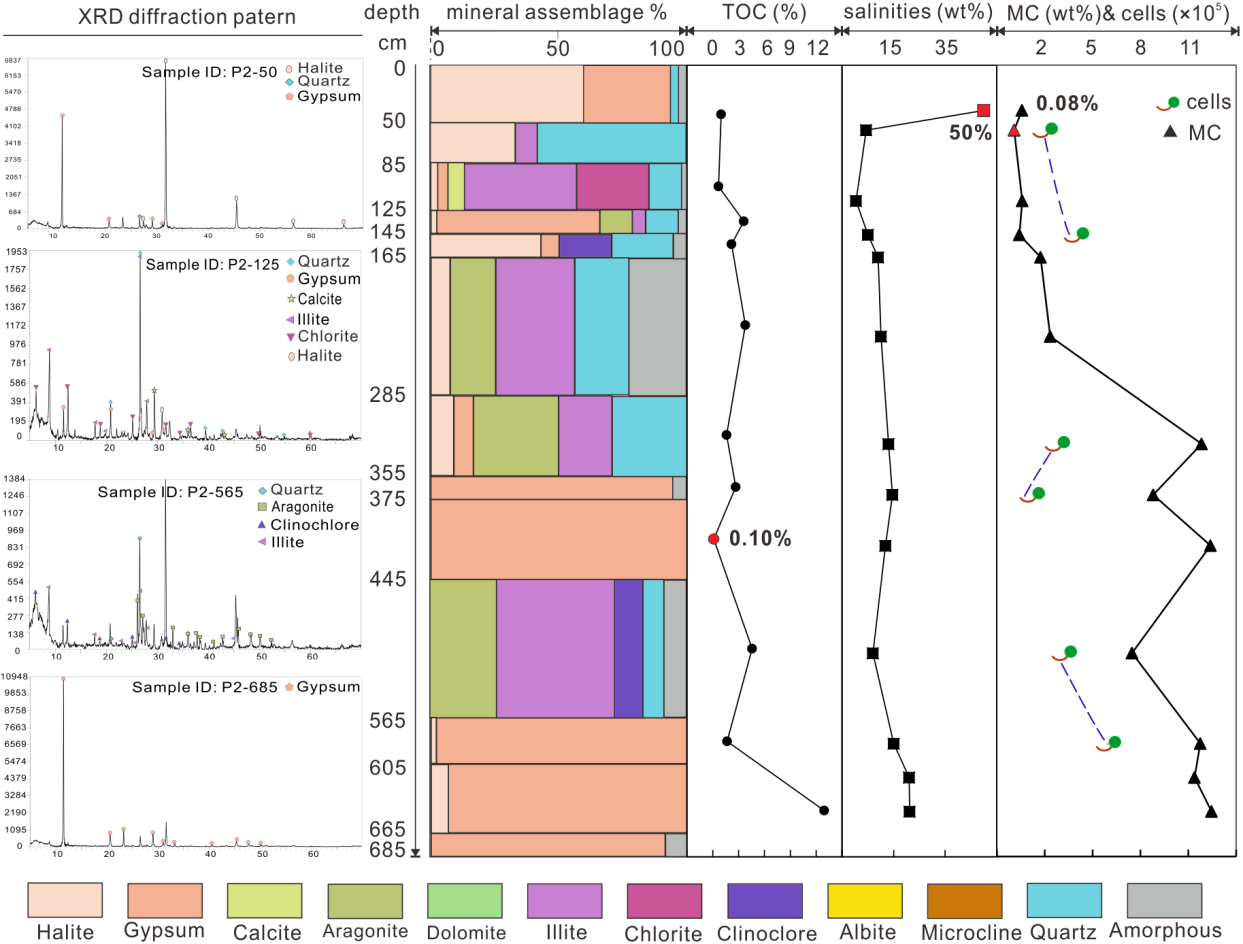
XRD diffraction patterns, mineral assemblage, TOC, salinity, moisture and cell numbers of P2 profile.

The TOC of profile P1 (Fig 2) ranged from 0.12% to 1.95%, while those of profile P2 (Fig 3) ranged from 0.10% to 12.95%. Salinities of P1 samples had a range between 7% and 50% and those of profile P2 varied from 0.4% to 50%. The highest salinity values in both profiles were in the uppermost layers. The moisture contents of the samples varied greatly along the profiles. In P1 profile the lowest moisture content was 0.33% in the uppermost layer, and the highest value 8.55% was located near the bottom (480–510 cm). For profile P2, the lowest moisture value 0.08% was near the surface (50–85 cm), and the highest value 12.45% was observed in the bottom sample.

In general, samples collected from both profiles were mainly composed of evaporites, fragments and other amorphous materials. For profile P1, the mineral assemblages changed with depth and four units could be identified. The first one was located at the 410–590 cm depth interval, which was dominated by phyllosilicates and carbonates. The second unit with a depth between 100 and 410 cm consisted mainly of sulfates and chlorides. Sediments between 37 and 100 cm contained more fragments such as albite and quartz. The fourth unit, 37 cm deep, was dominated by chlorides with a minor fraction of sulfates. As for profile P2, five units could be identified according to their mineral composition. The first unit, from 565 to 685 cm in depth, was mainly composed of sulfates. The second unit, from 445 to 565 cm consisted of carbonates and detrital minerals. The third unit, from 355 to 445 cm, was dominated by gypsum. The forth unit, from 85 to 355 cm, contained carbonates and phyllosilicates. The top unit, from 85 cm to the surface, consisted mainly of chlorides.

### Identification of microbial isolates

A total of 50 isolates of aerobic heterotrophic bacteria and 7 fungal isolates were obtained from 22 out of 23 samples along the two profiles and 3 out of 7 from surface samples. No archaea has been isolated in this study.

Bacterial isolates showed ≥97% 16S rRNA gene sequence homology with members of the *Bacillus* (major species, 25 strains), *Oceanobacillus*, *Halobacillus*, *Gracilibacllus*, *Thalassobacillus*, *Sediminibacillus*, *Ornithinibacillus*, *Pontibacillus* genera affiliated within the Bacillales order belonging to the Firmicutes phylum, and *Microbacterium* and *Nocardiopsis* genera affiliated within Actinomycetales and Streptosporangiales orders respectively belonging to the Actinobacteria phylum. Fungal isolates showed ≥ 98% ITS1 sequence homology with members of the *Aspergillus* and *Penicillium* genera of the Eurotiales order and the *Cladosporium* genera affiliated within the Capnodiales order, all of them of the Ascomycota phylum, which is the most representative of the xerophilic fungi [71].

**Fig 4:**
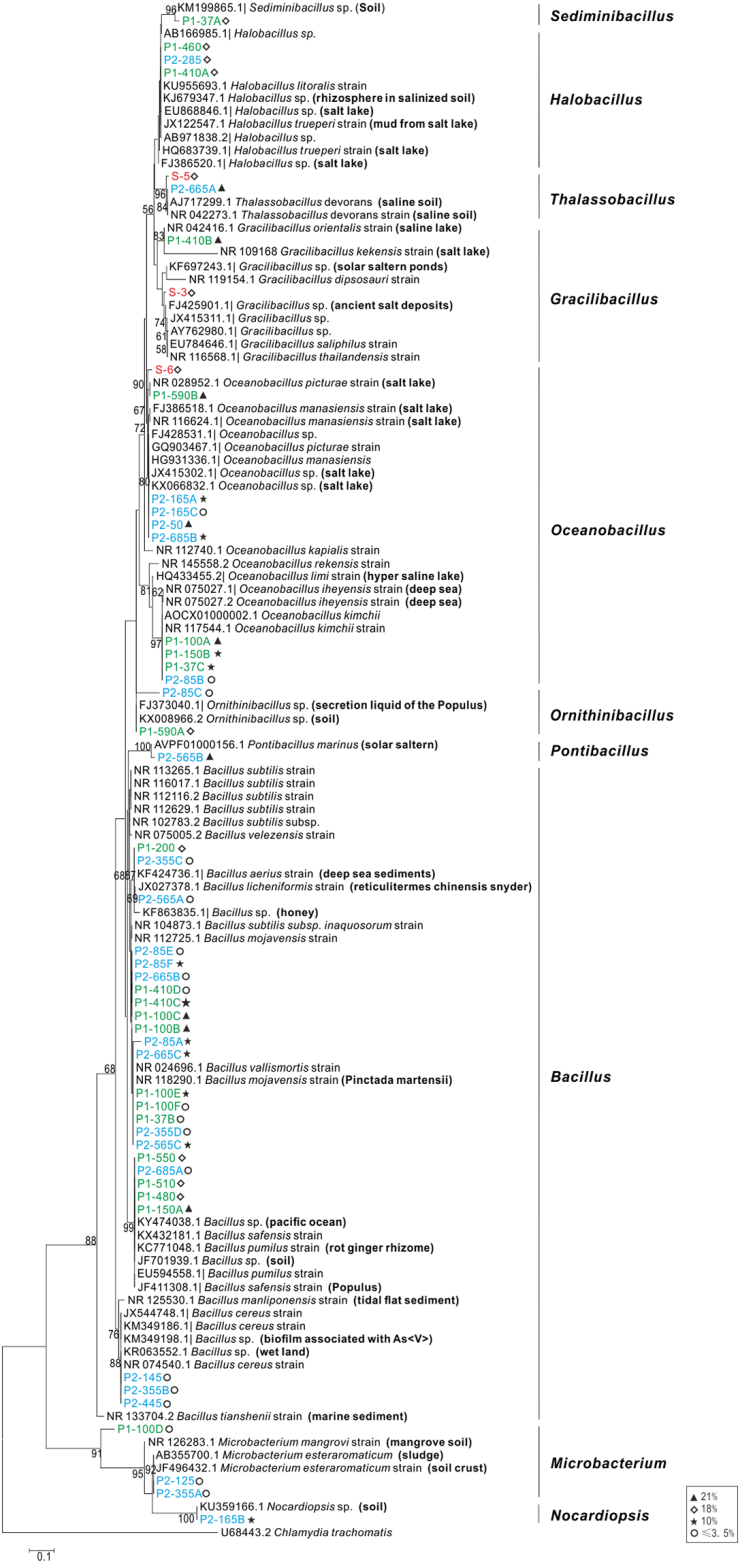
Phylogenetic dendrogram of culturable bacteria from Dalangtan Playa based on 16S rRNA gene sequences.

**Fig 5:**
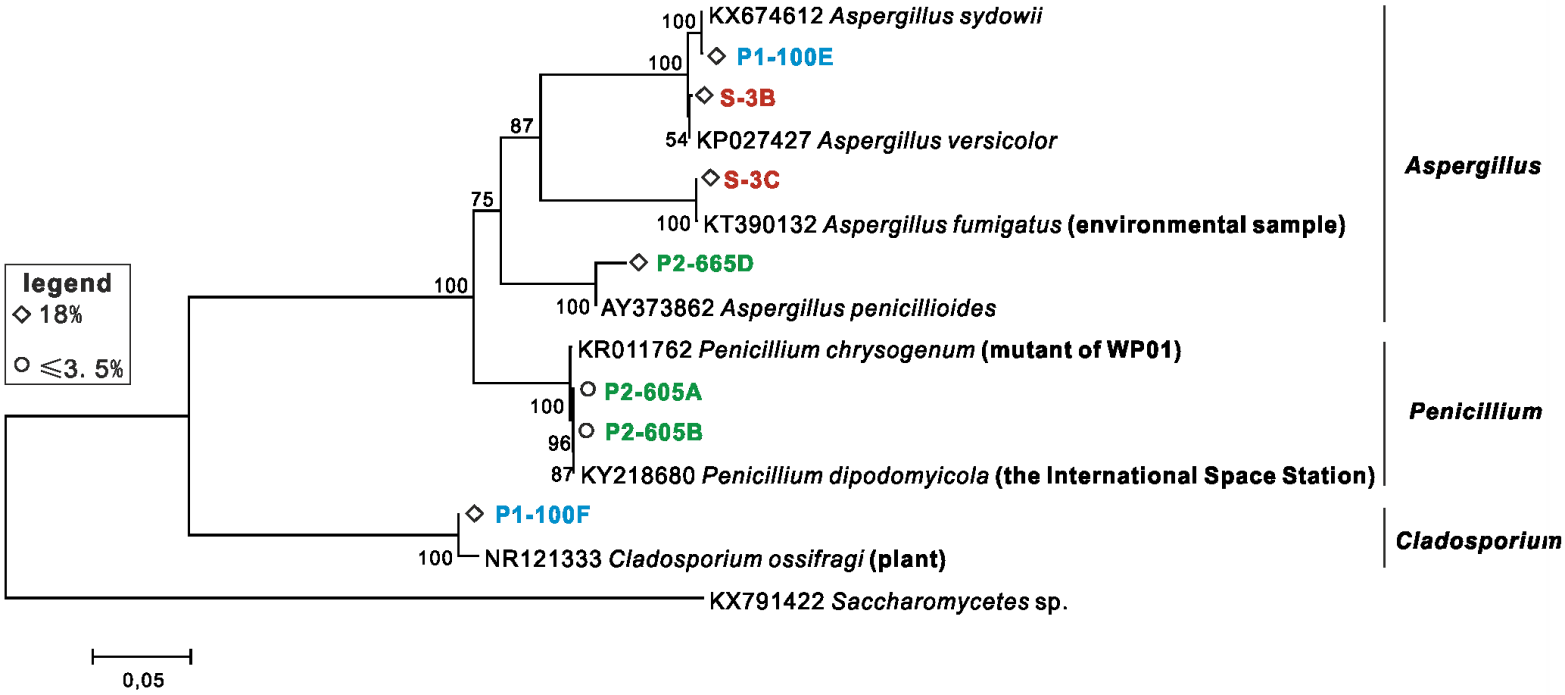
Phylogenetic dendrogram of culturable fungi based on rRNA-ITS gene sequences in Dalangtan Playa.

Phylogenetic trees based on the 16S rRNA and 18S ITS1 sequence comparison were generated for bacterial (Fig 4) and fungal isolates (Fig 5) respectively.

Maximum Likelihood algorithm with a T92+G nucleotide substitution model; 1000 bootstrap replicates were performed and values with >50% are shown in the tree. Sequences obtained in this study are in bold, and red, green and blue denote surface samples, subsurface samples P1 and P2 respectively. Salinities of the media on which isolates were obtained are marked in the figure.

Maximum likelihood algorithm with a K2+G nucleotide substitution model; 1000 bootstrap replicates were performed and values with >50% are shown in the tree. Sequences obtained in this study are in bold, and red, blue and green denote surface samples, subsurface samples P1 and P2 respectively.

Among the 50 bacterial isolates, 9 isolates grew with a 21% salinity (Fig 4): 3 *Oceanobacillus*, 3 *Bacillus*, 1 *Gracilibacillus*, 1 *Thalassobacillus* and 1 *Pontibacillus*. Twelve were isolated with a salinity of 18%: 4 *Bacillus*, 3 *Halobacillus*, 1 *Oceanobacillus*, 1 *Gracilibacillus*, 1 *Thalassobacillus*, 1 *Ornithinibacillus* and 1 *Sediminibacillus*. And 11 were cultured in 10% salinity media: 6 *Bacillus*, 4 *Oceanobacillus* and 1 *Nocardiopsis*. Twelve *Bacillus*, 3 *Microbacterium*, 2 *Oceanobacillus* and 1 *Ornithinibacillus* were cultured with ≤ 3.5% salinity media. As for fungal isolates, five were cultured with a salinity of 18% (Fig 5): *Aspergillus sydowii*, *A*. *versicolor*, *A*. *fumigatus*, *A*. *penicillioides* and *Cladosporium*. Two *Penicillium* strains were isolated with a 3% salinity media.

Among the isolated strains, sporulation and moderately halophilic genera belonging to the *Bacillus* and *Oceanobacillus* were present in most of the samples. In addition, pigments were observed from pure cultures of *Bacillus licheniformis* (green, P2-565A), *Mycobacterium esteraromaticum* (green, P2-355A and P2-125), *Microbacterium* (pink, P1-100D), *Halobacillus trueperi* (pink, P2-285) and *Cladosporium ossifragi* (black, P1-100A).

**Fig 6:**
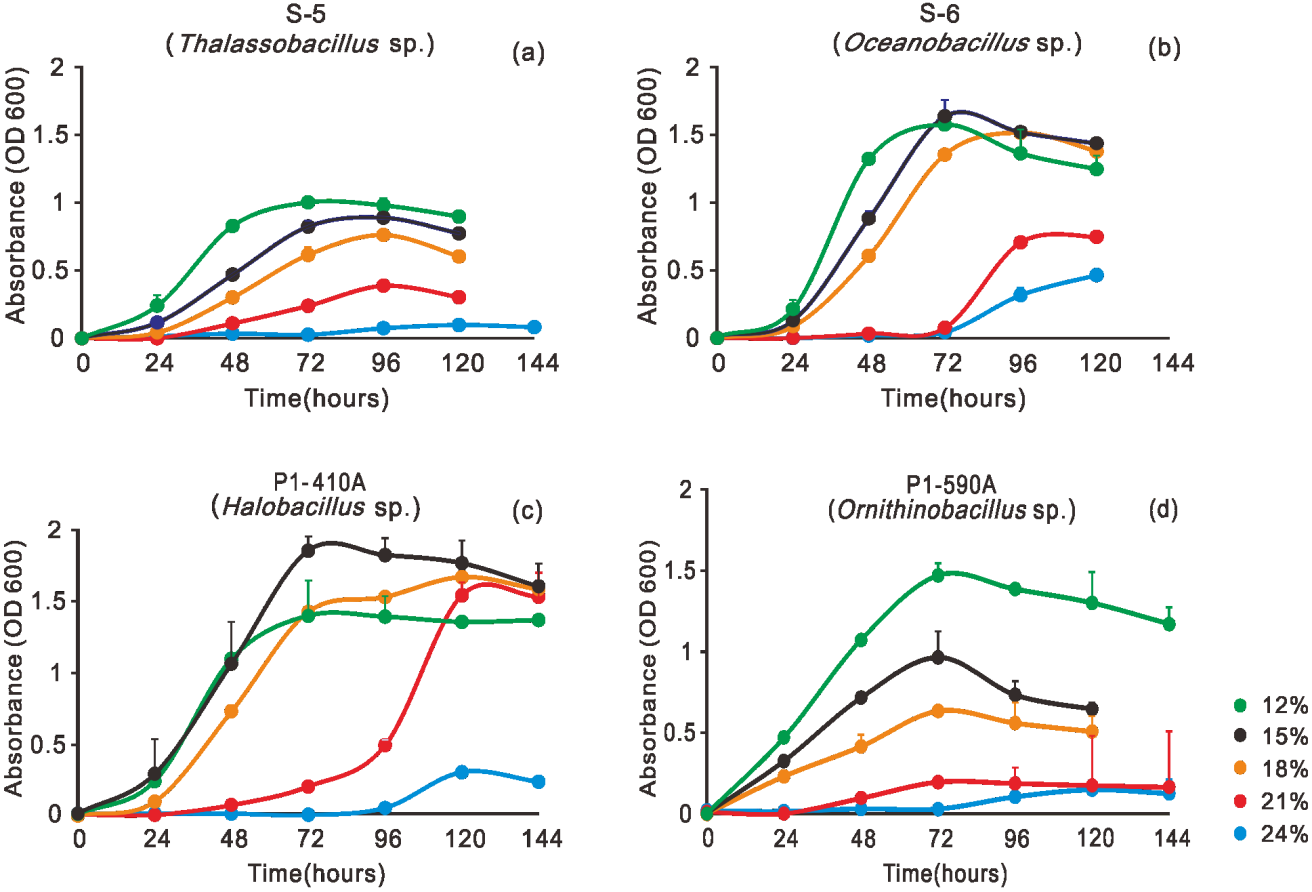
Growth curves of four bacterial isolates under different salinities ranging from 12% to 24%.

Four bacterial strains isolated from media with a salinity of 18% were tested for their growth at salinities ranging from 12% to 24%. Specifically, *Oceanobacillus* sp. (S-6) and *Halobacillus* sp. (P1-410A) showed an optimal growth at 15% salinity, while *Thalassobacillus* sp. (S-5) and *Ornithinibacillus* sp. (P1-590A) preferred a salinity of 12% (Fig 6).

Most isolates showed a rod-like shape with 0.5–5 μm in length (S1 Fig). The cells of *Microbacterium* (S1b Fig) have relatively smaller size (length of 0.5–1.5 μm) than the rest. *Gracilibacillus* present a “matchstick-like” morphology (S1c Fig). Micro particles were observed spreading around the cells of *Bacillus*, *Oceanobacillus* and *Halobacillus* (black arrows in S1a, S1d and S1e Fig). Cells of *Sediminibacillus* are lanky in shape with a length of about 4 μm (S1f Fig).

### Microbial community abundance analyzed by culture independent methods

Twelve environmental samples were subjected to DNA extraction strategy. The concentration of extracted DNA of the samples was between 1.0 to 5.0 ng/μl. Amplification using archaeal (Arc 1F, 1R), bacterial (21F, 1492R and S-D-Bact-0341-b-S-17, S-D-Bact-0785-a-A-21) and eukaryal (563F, 1132R) primers did not give positive results as indicated by gel electrophoresis of PCR products. Meanwhile, test with *E*. *coli* as control showed that no amplification inhibitors were present in the extracted DNA samples.

**Fig 7:**
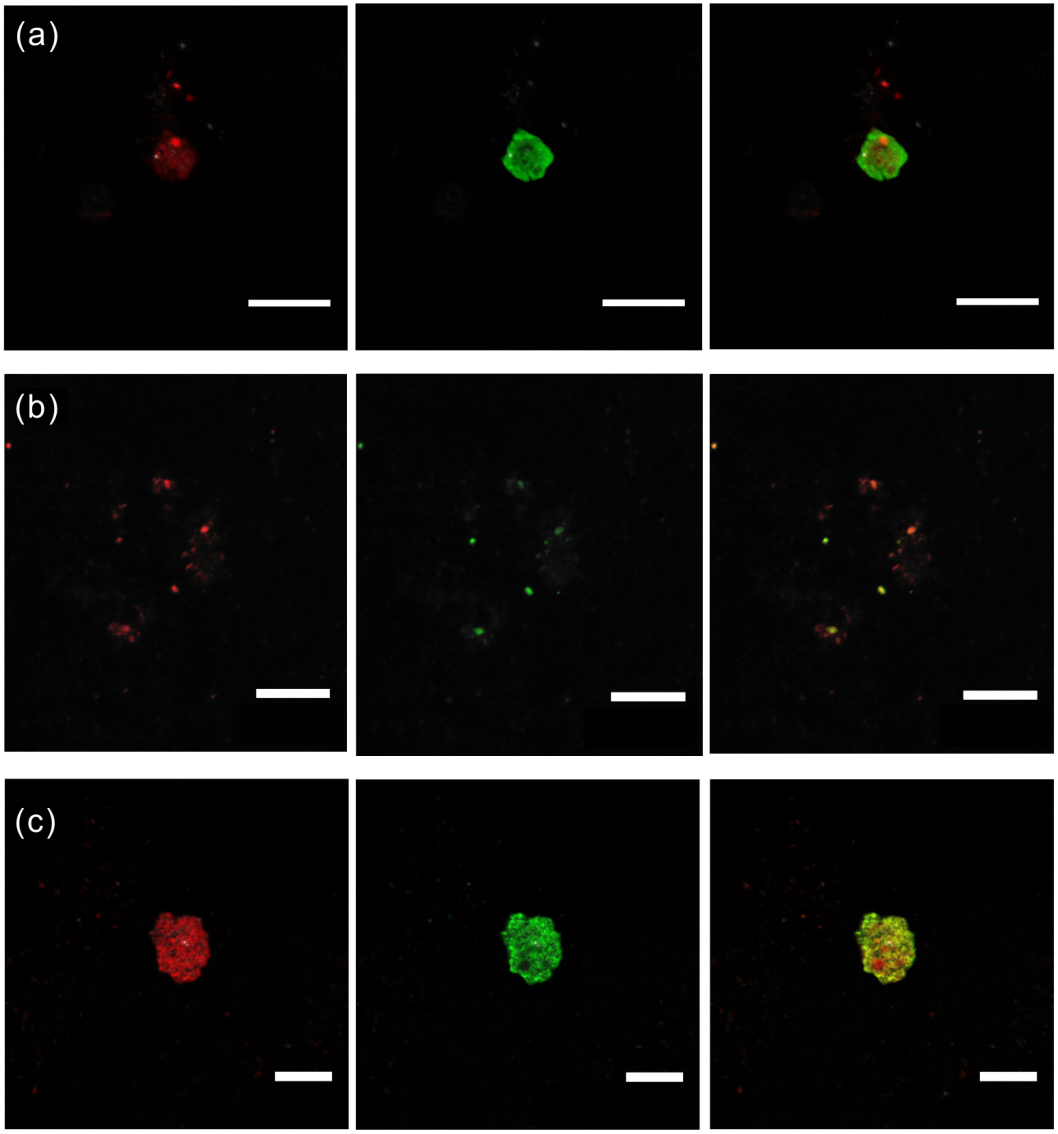
CARD-FISH hybridization analysis of subsurface samples from Dalangtan Playa. (a) for P1-200, (b) and (c) for P2-285. From left to right: epifluorescence micrographs showing bacterial cells hybridized with the universal probe EUB388 I-III mix (left column), the same field stained with Syto9 (middle column), and the overlap of stained field (right column). Scale bar: 10 μm.

CARD-FISH and DNA stains were applied for the identification and quantification of the entire indigenous microbial community in the original sediments. Bacterial clusters were efficiently hybridized by CARD-FISH using the EUB 388 I-III mix probe (Fig 7). A total of (1.54±0.49) ×10^5^ bacteria g^−1^ in average were counted for subsurface samples of P1 and (3.22±0.95) ×10^5^ bacteria g^−1^ for those of P2. Counted cell numbers of individual samples were shown in Figs 2 and 3 accordingly. Meanwhile, hybridization with Firmicutes specific probe indicated that 76.6% and 75.7% of bacteria belong to this phylum. No signals using the archaeal probe were detected.

## Discussion

Of all the terrestrial saline flats, Dalangtan Playa shows unique characteristics when compared with others. Most of the terrestrial saline flats are located in regions of maximum aridity [72] such as Nabta Playa in Egypt, Racetrack Playa in the US, Petregan Playa in Iran and Salar Grande in Chile. For example, the Salar Grande in Atacama Desert, 640–750 m above sea level, is filled with almost unaltered pure halite [35] with 0.4% TOC in the halite crusts [73]. In contrast, Dalangtan Playa is an inland saline depression with a higher altitude of over 2700 m, which receives stronger UV radiation [74] with an annual mean solar radiation of over 6800 MJ/m^2^ [75]. The monthly surface downwelling shortwave radiation (rsds, containing UV radiation) in Dalangtan area (38.5 N, 91.5 E) was 266.6 KJ/m^2^·S (data during July of 2013), while at the Salar Grande area (21.5 S, 70.5 W), it was 124.3 KJ/m^2^·S (http://ceres.larc.nasa.gov/). It is well known that UV radiation can damage microbial DNA and results in heritable changes via mutagenesis, increasing genetic variation [76]. The strong UV radiation at Dalangtan Playa is severely challenging to the survival of microbes. Moreover, one of the most important food sources for microorganisms, organic carbon is very limited in the surface sediments of Dalangtan Playa with an average TOC of 0.03%. All these extreme conditions provide an ideal model as terrestrial Mars analogue with astrobiological implications.

Molecular amplification of microbial DNA from samples collected from Dalangtan Playa is full of challenges and no efficient genomic data has been obtained so far. In addition, bacterial numbers in this environmental sediment are ~10^5^ g^-1^as indicated by CARD-FISH analysis, which is comparable to those in Yungay soil of Atacama [34].

Despite the low numbers of cultivable microorganisms recovered in Dalangtan Playa samples, we have been able to reveal a more diverse microbial community using different media and various salinities. Contamination is unlikely because no microbial growth was observed on control plates. Moreover, ≥70% of the isolates are affiliated with others from similar hyper saline conditions. Specifically, bacterial isolates from surface samples S-3 and S-5 showed a 97% and 99% identity with *Gracilibacillus* sp. (FJ425901) and *Thalassobacillus* sp. (AJ717299) respectively, which had been isolated from ancient salt deposits (NCBI) and the Great Salt Plain [77]. The isolate from S-6 was affiliated with *Oceanobacillus* sp. (JX415302) which was reported previously in a salt lake (NCBI). Bacteria isolated from the subsurface samples show high identity (≥98%) with bacterial strains isolated from hyper-saline environments such as saline soil (*Thalassobacillus* sp. NR042273) [78], salt lakes (*Halobacillus* sp. EU868846) [79], deep-sea sediments (*Bacillus aerius* strain, KF424736) (NCBI), rhizosphere of plants in salinized soil (*Halobacillus* sp., KJ679347) (NCBI) and soil crust (*Microbacterium esteraromaticum* strain, JF496432) (NCBI). Some were isolated from peculiar environments such as a biofilm associated with As(V) (*Bacillus* sp., KM349198) and liquid secretions of *Populus* (*Ornithinibacillus* sp., FJ373040) (NCBI). One of closest fungal references *P*. *dipodomyicola* (KY218680), was isolated from the International Space Station (NCBI). Furthermore, the microbial assemblages in Dalangtan area are dominated by members of the Firmicutes, Actinobacteria and Ascomycota phyla, similarly to those detected in the arid Atacama Desert [80, 81]. All of these strengthen the evidence for the fidelity and reliability of our data.

Several factors such as RH and TOC may control fungal colonization in intense radiation conditions. For example photosynthetic algae and cyanobacteria and photoprotective pigment-forming fungi [82] were only detected within the microenvironment with RH ≥60% in the gypsum crust on the surface of Atacama [83]. In this study, only S-3, one surface sample out of seven, yielded fungal isolates (*Aspergillus*). The gray-white S-3 sample was collected under a light brown salt crust (Fig 1a) which might protect fungi from UV damage, favoring their survival. In addition, S-3 contains 71.9% halite and 11.4% gypsum. The hygroscopic properties of halite may contribute to deliquescence generating a wet microenvironment for microorganisms, which needs further study to prove it. Moreover, S-3 was also characterized by the highest content of detrital grains (i.e. quartz, 16.7%) and TOC (0.12%) of the 7 selected surface samples. Given the heterotrophic life of fungi, the high TOC content in S-3 may also contribute to the recovery of *Aspergillus*. Besides that, *Aspergillus* was also isolated from 2 subsurface samples in our study. However, the mineralogy of each of these two subsurface samples is quite different, from each other, as well as from S-3 sample. P1-100 is dominated by detrital grains containing albite, quartz, and a small portion of evaporite minerals and the black pigment-forming fungal isolate *Cladosporium* (P1-100A) as well. It has the second highest moisture content of 2.94% along profile P2. P2-665 has a relatively high moisture content of 11.39%, which may contribute to the recovery of *Aspergillus*. In addition, two *Penicillium* strains were isolated from the sample of P2-605 with high moisture content (11.76%). Collectively, samples with high TOC content or high moisture may favor fungal recovery, but results of microbial recovery showed no obvious preference for mineral assemblage.

Indeed, the availability of liquid water is the most critical constraint for life [36]. Most cellular systems of known life forms on Earth are active within the range of 1 to 0.900 water activity [84]. And 0.605 water activity has been considered as the low limit for microbial growth [85], until Stevenson, Hamill [86] reported recently that the lowest water activity for fungal germination (*Aspergillus penicillioides*) was 0.585. Although the water activity was not measured in our study, the moisture content to some degree also can indicate the water availability in samples. In general, cell numbers of bacteria have a positive relationship with moisture contents (indicated from Figs 2 and 3, the last columns). And relative high moisture contents (11.39 to 12.45%) in samples of P2-685, P2-665, P2-605 and P2-445 favor for the recovery of bacterial and fungal isolates.

Among all bacterial isolates, *Bacillus* was the most widely spread genus. They were recovered from samples at different depths with variable physicochemical properties. For example, the salinities from 5 to 50%, the moisture contents from 0.08 to 12.45% and the TOC values from 0.10 to 12.95%. Moreover, *Bacillus* showed no specific preference for mineral assemblages. The mineral compositions of samples with bacterial isolations varied from gypsum (9 out of 15 samples), carbonates associated with clay or detrital grains (5 samples), chlorides (4 samples) and mixtures of evaporites (including halite) and detrital grains. Among the 50 bacterial isolates, 46 strains are affiliated to Firmicutes (92%), which was supported by CARD-FISH quantification of Firmicutes to be 56–95% of total bacteria.

*Bacillus* can form endospores under harsh conditions, which are strongly resistant to UV radiation and desiccation. It has been reported that under extreme desiccation and high vacuum condition (10^−6^ Pa at 77 K for 24 h), 55% of *Bacillus subtilis* spores are still able to germinate. Dormant spores of various *Bacillus* species, including *B*. *subtilis*, are 5 to 50 times more resistant to UV radiation than the corresponding growing cells [87]. In fact, due to their properties, members of *Bacillus* genus are considered as targeted microorganisms both for astrobiology and for planetary contamination preventive strategies. It has been reported that, 66% of 358 strains of cultivable microorganisms collected from the surface of Mars Science Laboratory (NASA) were members of the *Bacillus* genus [88]. Several studies on *Bacillus* exposed to simulated Mars environmental conditions have been conducted. Under simulated Martian atmospheric pressure and composition condition for 19 days, spores of *B*. *subtilis* had impairment for germination but similar survival rates than those under Earth conditions [89]. When exposed to simulated Mars Solar Radiation (254-nm UV light), B. subtilis had retained the possibility to initiate germination-associated metabolic processes and to produce biological signature molecules [90]. Moreover, under multiple simulated factors (high UV, low pressure, low temperature and CO_2_ atmosphere) for 24 h, 16.7% of *B*. *subtilis* still exhibited positive growth [91]. In addition, *B*. *pumilus* spores were able to grow in the dark after 18-month’s exposure to both space and Mars simulated conditions [92]. Due to the strong resistance to extreme conditions, spore-forming bacteria can withstand space environmental conditions. Liquid water is required for spore germination and subsequent growth. If present on extraterrestrial planets (e.g., from deliquescent evaporites or brines), spores of *Bacillus* can germinate despite of the high salt concentrations [93]. All these studies strongly support the interest of *Bacillus* in relation to life exploration on Mars particularly in evaporites which formed under saline conditions.

Investigation of microbial communities in terrestrial saline deposits are of astrobiological importance considering the abundance of evaporites on Mars [94, 95]. Many evaporitic minerals including carbonates [96], hydrated sulfates [56], phyllosilicates [3] and chlorides [97] have been detected or identified on Mars. Some of them are proved to have an ancient Martian lacustrine/playa setting similar to Dalangtan Playa, e.g. the “bathtub rings” in the Hellas basin indicates it was filled by water [98]. Interior layered deposits (ILDs) in Juventae Chasama (Fig 1b’) are covered by hydrated sulfates (e.g. kieserite <MgSO_4_·H_2_O> and szomolnokite <FeSO_4_·H_2_O >). It is proposed that they deposited in a lacustrine setting [99] though more complicated progresses might be involved in Martian history [100]. The layered deposits in Dalangtan Playa are composed of sulfate and chloride on the surface (Table 1). Kieserite (MgSO_4_·H_2_O) has been reported before [52]. And in the vertical profile, evaporites precipitated from ancient lake are identified (Figs 2 and 3). These evidences prove that the layered deposits in Dalangtan Playa are good analogue to ILDs in Juventae Chasma both morphologically and mineralogically.

In addition, a kind of Martian surface dust, which is mixed with and indurated by Ca/Mg-bearing sulfate salts, has been observed on Mars [101]. Meter-scale deep profiles could be found on Earth as good geochemical and physical analogues. Playas have also been identified on Mars as sedimentary structures by the MER Opportunity (NASA, 2003) landing area in the Endurance crater. Especially, Burns formation, a ~7 m thick sedimentary rock, was interpreted to represent a succession of reworked impure evaporitic sandstones [102]. Evaporite minerals such as sulfates (accounting for 35–40%) and chlorides (≤2%) were identified at the opening profile. And geological history inversion suggested that there was a record of a playa lake, which was deposited by a mixing of basaltic and evaporative mud. Afterward, the desiccated playa lake was modified by aeolian and minor subaqueous processes [103]. Dried from a lake, located in aeolian affected region, Dalangtan Playa is a counterpart of Burns formation on Earth in terms of geological background. Since Mars has experienced cooling [104] and drying [105] processes, knowledge on the evaporation deposits in Dalangtan Playa could assist by comparison to understand Mars near surface geological history. Deeper research on Mars evaporitic or ancient lacustrine conditions by space missions together with analogous and microbiological studies on their terrestrial counterpart could facilitate the detection of past or extant life on Mars.

## Conclusion

Dalangtan Playa is an important analogue research site for Martian life exploration. A relatively low microbial diversity (10^5^ bacteria g^-1^) was indicated by both isolation and culture independent methods. Both bacteria and fungi were isolated from Dalangtan Playa where colonization was controlled mainly by moisture content and TOC of samples. The halotolerant (e.g. *Oceanobacillus*, *Halobacillus*) and spore-forming *Bacillus* genera dominate the cultivable bacterial communities which is consistent with the observed quantification result of Firmicutes (56–95%) in our samples and the remarkable ability of *Bacillus* to survive under conditions with multiple extreme factors. Our results suggest that, for the exploration of life traces on super arid Mars, especially in ancient lacustrine conditions, evaporitic microenvironments may provide refuge for life colonization, and halotolerant spore-forming microorganisms seem to be excellent candidates for this lifestyle. Further studies on Dalangtan Playa using microbial life detection techniques should be applied in further extraterrestrial life exploration missions.

## Supporting information

S1 Fig SEM images of bacterial isolates from Dalangtan Playa cultured in the modified growth media (MGM) with salinities of 18% (a, c, d, e, and f) and 3% (b). (TIF)

S1 Table Ingredients of Arq medium used for archaeal isolation in this study. (DOCX)
