# Supplementary figures and images for: Dalangtan Playa (Qaidam Basin, NW China): Its microbial life and physicochemical characteristics and their astrobiological implications
Source: PLoS One. 2018 Aug 1;13(8):e0200949. doi: 10.1371/journal.pone.0200949 (PMC6070256; doi:10.1371/journal.pone.0200949)

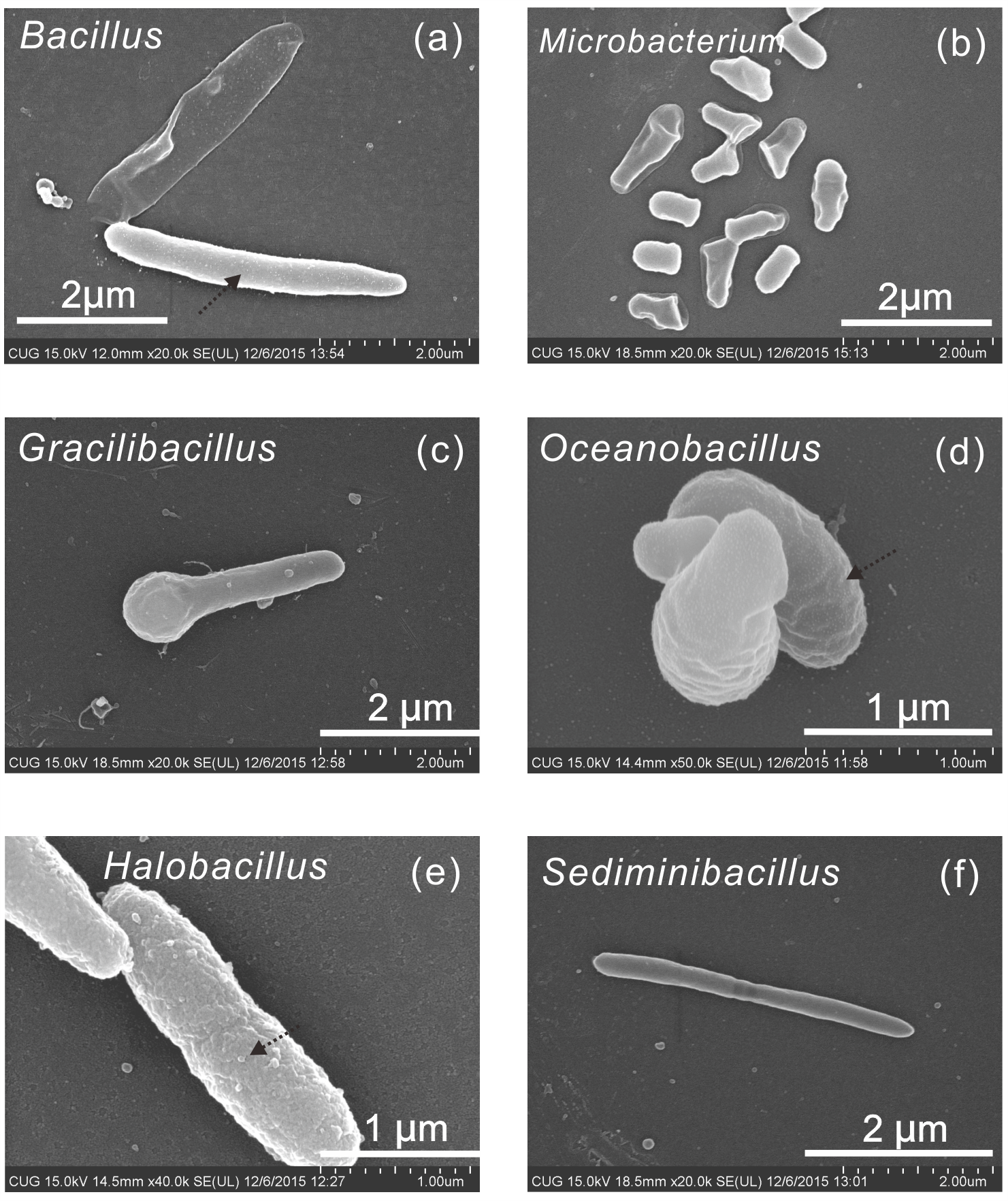

Supplement: S1 Fig — (TIF) [file pone.0200949.s001.tif]
