# Supplementary material for: Dalangtan Playa (Qaidam Basin, NW China): Its microbial life and physicochemical characteristics and their astrobiological implications
Source: PLoS One. 2018 Aug 1;13(8):e0200949. doi: 10.1371/journal.pone.0200949 (PMC6070256; doi:10.1371/journal.pone.0200949)
Supplement: S1 Table — (DOCX) [file pone.0200949.s002.docx]

**S1 Table. Ingredients of Arq medium used for archaeal isolation in this study.**

| pH=8 adjust with 1M NaOH | in 1 liter (g) |
| --- | --- |
| NaCl | 174 |
| MgCl_2_.6H_2_O | 30 |
| MgSO_4_.7H_2_O | 45.4 |
| CaCl_2_.2H_2_O | 0.8 |
| KCl | 4.5 |
| NaHCO_3_ | 0.16 |
| NaBr | 0.5 |
| Yeast extract | 5 |
| Casamino acids | 0.5 |
